# Association between systemic immune-inflammation index(SII) and all-cause and cardiovascular mortality in heart failure patients: a single-center retrospective analysis

**DOI:** 10.3389/fcvm.2026.1823641

**Published:** 2026-04-24

**Authors:** Junlan Zhang, Nan Lyu, Yaping Zhang, Pan Chen, Qianyu Ma, Nana Hu, Bingxin Men, Xiaolei Shi, Changsen Wang, Jin Zhang

**Affiliations:** 1 Lanzhou University First Hospital, Lanzhou, China; 2 Wenzhou Medical University School of Public Health, Wenzhou, China; 3 Anhui Medical University Affiliated Fuyang People's Hospital, Fuyang, China

**Keywords:** all-cause mortality, cardiovascular mortality, heart failure, inflammation, systemic immune-inflammatory index (SII)

## Abstract

**Objective:**

This study aimed to investigate the association between the systemic immune-inflammatory index (SII) and mortality in patients with heart failure (HF).

**Method:**

We conducted a retrospective cohort study of 1,084 HF patients. In this retrospective cohort study, we enrolled patients hospitalized for heart failure between January 2022 and June 2023. Follow-up was conducted via telephone and outpatient visits until death or July 22, 2025, with all-cause and cardiovascular mortality as primary endpoints. Patients were categorized by log-transformed SII (LnSII). Cox models assessed associations between LnSII and mortality, while restricted cubic splines evaluated nonlinearity. Subgroup, mediation (NT-proBNP, LVEF), and sensitivity analyses were performed.

**Results:**

A higher LnSII was significantly associated with an increased risk of all-cause mortality (fully adjusted HR = 1.59, 95% CI: 1.03–2.46), No statistically significant association was detected with cardiovascular mortality, which may be attributable to the limited number of cardiovascular deaths (*n* = 60) and consequent reduced statistical power. Subgroup analysis revealed a significant interaction with smoking status (*P* for interaction=0.023), showing a stronger association between LnSII and all-cause mortality among smokers (HR = 2.41, 95% CI: 1.57–3.68). Mediation analysis indicated that NT-proBNP and LVEF mediated 35.8% and 15.0% of this association, respectively.

**Conclusion:**

Elevated SII is independently associated with an increased risk of all-cause mortality in HF patients, particularly among smokers, and may serve as a useful prognostic biomarker.

## Introduction

Heart failure (HF) represents the terminal stage of various cardiovascular diseases, significantly impairing quality of life. According to the 2024 Summary of China's Cardiovascular Health and Disease Report, the incidence of adult HF remains high. Monitoring data from 2023 indicates that the non-recovery discharge rate (in-hospital death or non-medically advised discharge) among hospitalized HF patients was 10.2%, with an in-hospital mortality rate of 2.6%, a non-medically advised discharge rate of 7.6%, and a 30-day readmission rate of 11.0% (1). Furthermore, the 5-year mortality rate after HF diagnosis reaches 50% (2), and the poor prognosis of HF imposes a significant healthcare burden on patients and the national healthcare system (3, 25). Therefore, strengthening the early detection and treatment of HF is essential to reduce patient mortality. Studies indicate that patients with systemic inflammatory diseases have a significantly higher risk of cardiovascular disease than the general population, suggesting that inflammatory variables can serve as predictive indicators for cardiovascular disease (4).

The Systemic Immune-Inflammation Index (SII) is a newly developed inflammatory biomarker combining lymphocyte, neutrophil, and platelet counts. Studies have confirmed that SII initiates a cascade of cardiac remodeling and promotes pathological changes in HF. However, the relationship between SII and outcome events in HF patients remains unclear (5). Therefore, further investigation into the association between SII and outcome events in HF patients is necessary to provide insights for precision management of HF patients.

## Materials and methods

### Study population

**Figure 1:**
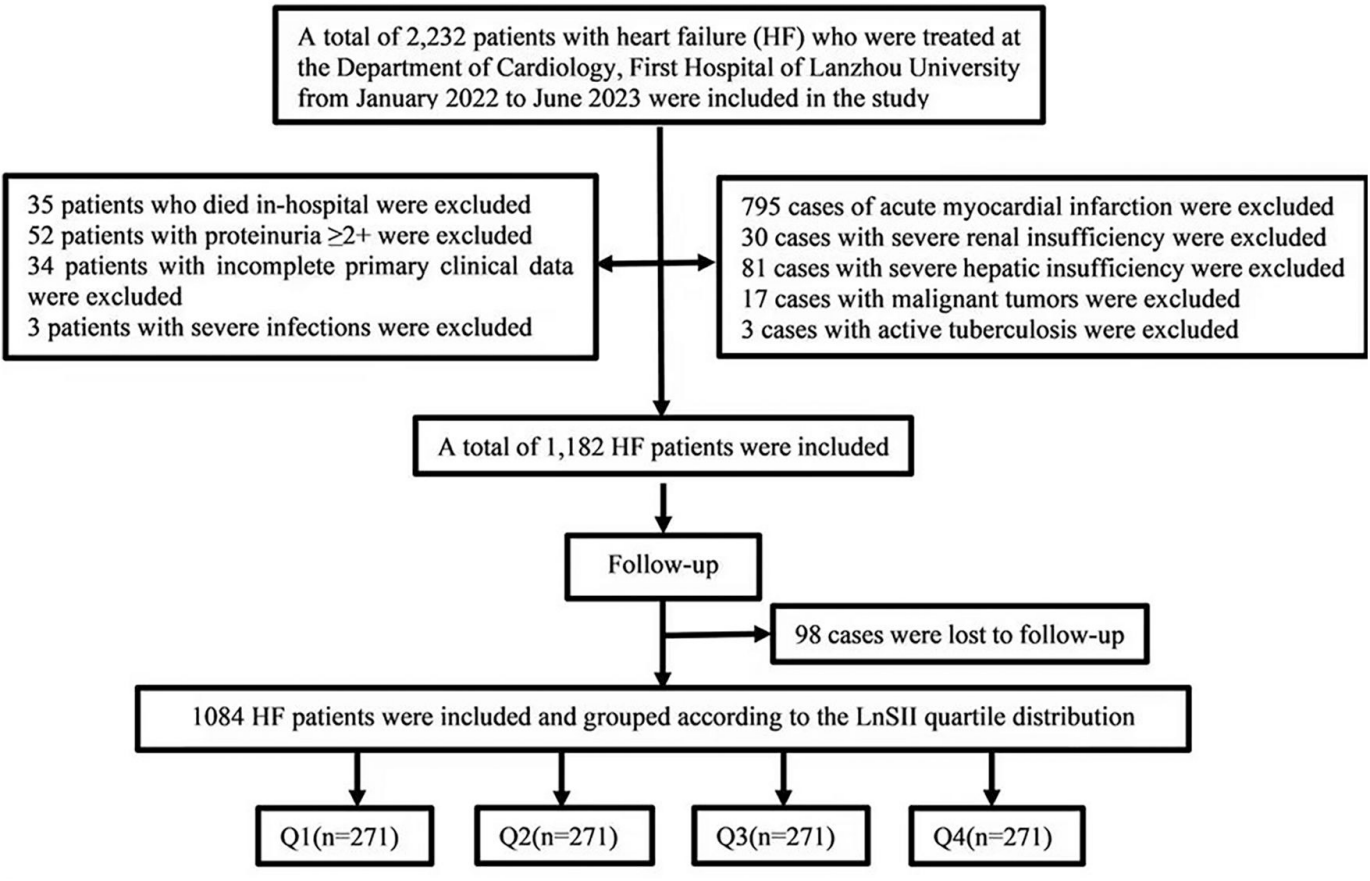
Researcher screening flowchart.

This retrospective study consecutively enrolled 2,232 heart failure patients admitted to the Department of Cardiology of a tertiary hospital in China between January 2022 and June 2023. After screening based on inclusion and exclusion criteria, 1,182 cases were identified. With 98 cases lost to follow-up, 1,084 cases were ultimately included. Inclusion criteria: (1) Age 18 years ≤age <90 years; (2) Patients meeting the diagnostic criteria for HEmrEF or HErEF as defined in the *2018 Chinese Guidelines for the Diagnosis and Treatment of Heart Failure* based on symptoms, physical findings, laboratory tests, and echocardiography, or patients with a history of heart failure and a left ventricular ejection fraction (LVEF) ≤ 49% at admission. Exclusion criteria: (1) Incomplete primary data; (2) Malignant tumors, severe infectious diseases, active tuberculosis, autoimmune diseases; (3) Pregnancy; (4) Severe hepatic or renal insufficiency, proteinuria (urine protein ≥2+); (5) In-hospital death; (6) Acute myocardial infarction. The screening process is illustrated in Figure 1.

### Clinical data collection

#### General information

Recorded patient age, gender, systolic blood pressure, diastolic blood pressure, heart rate, height, weight, smoking history, history of hypertension, history of diabetes, history of stroke, prior percutaneous coronary intervention, prior myocardial infarction, cardiomyopathy, valvular heart disease, and comorbidities (including concomitant atrial fibrillation or atrial flutter, ventricular arrhythmias), medication history (β-blockers, angiotensin-converting enzyme inhibitors, angiotensin receptor blockers, angiotensin-neprilysin inhibitors, aldosterone receptor antagonists, sodium-glucose cotransporter 2 inhibitors, antiplatelet agents, anticoagulants), echocardiographic parameters [left ventricular ejection fraction (LVEF), left ventricular end-diastolic volume, left ventricular end-systolic volume], NYHA functional class (I,II,III,IV) and calculate body mass index.

#### Laboratory tests

Within 24 h of admission, obtain the following parameters using standard laboratory methods: white blood cells, red blood cells, hemoglobin, platelets, lymphocytes, monocytes, neutrophils, total cholesterol, triglycerides, high-density lipoprotein, low-density lipoprotein, lipoprotein a, N-terminal pro-B-type natriuretic peptide (NT-proBNP), Glomerular Filtration Rate (GFR) and calculate SII. SII (×10^9^/L) = Platelet count (×10^9^/L) × Neutrophil count (×10^9^/L)/Lymphocyte count (×10^9^/L).

#### Study subject grouping

To approximate a normal distribution for SII values, natural logarithms (LnSII) were taken and subjects grouped by quartiles.

### Follow-up and endpoint event definition

All patients were followed up via telephone and outpatient records starting from hospital discharge. Follow-up ended upon death or reached a cutoff date of July 22, 2025. Endpoint events were defined as: All-cause mortality: Total deaths due to any cause after hospital discharge. Cardiovascular mortality: Deaths due to acute myocardial infarction, sudden cardiac death, decompensated heart failure, stroke, or other cardiovascular causes (e.g., aortic dissection).

### Statistical methods

Statistical analysis was performed using SPSS 26.0 and R 4.5.1 software. Normally distributed variables were expressed as mean ± standard deviation, with intergroup comparisons using independent samples *t*-tests. Non-normally distributed variables were presented as median (P25, P75), with intergroup comparisons using the Kruskal–Wallis H-test. Categorical variables were expressed as percentages, with intergroup comparisons using chi-square tests or Fisher's exact tests. Cox proportional hazards models were employed to investigate the association between baseline LnSII and all-cause or cardiac mortality, with results presented as hazard ratios (HR) and 95% confidence intervals (CI). The proportional hazards assumption for the Cox models was assessed by testing the correlation between Schoenfeld residuals and survival time. A global test *P*-value > 0.05 was considered indicative of no violation of the assumption. To assess the stability of the primary findings against sampling variability, we performed bootstrap resampling with 1,000 iterations. In each iteration, the Cox proportional hazards model (Model 4) was refitted, and the hazard ratio (HR) for the highest versus lowest LnSII quartile (Q4 vs Q1) was estimated. Bias-corrected 95% confidence intervals were derived using the percentile method. Confounders were selected based on prior knowledge. To avoid over-adjustment bias, variables potentially located on causal pathways (LVEF and NT-proBNP) were excluded from the primary analysis model. Restricted cubic splines (RCS) were used to assess potential nonlinear associations. Subgroup analyses compared differences and interactions of variable factors on outcome events. Mediation analysis using the Bootstrap method (Mediation package) explored the potential mediating roles of NT-proBNP and LVEF in the relationship between LnSII and all-cause mortality and cardiac mortality. Sensitivity analyses tested the robustness of results. *P* < 0.05 was considered statistically significant.

## Study results

### Comparison of baseline characteristics Among HF patients in different LnSII groups

This study included 1,084 HF patients. With a mean follow-up of 29.3 months (range: 2 days to 39 months), 142 all-cause deaths (13.1%) and 60 cardiac deaths (5.5%) were observed. The cohort comprised 853 men (78.7%) and and 231 women (21.3%). The mean age was 62 years. Compared with patients in the lowest quartile (Q1), those in the highest quartile (Q4) of LnSII were older [median (interquartile range): 66 (57.00, 72.00)], had higher prevalence of stroke and hypertension (52.8%), lower smoking history (36.5%), lower LVEF [39 (34, 45)], and faster heart rate [86 (75, 103)]. Regarding laboratory parameters, patients in the highest LnSII quartile exhibited significant differences in NT-proBNP, white blood cells, red blood cells, hemoglobin, platelets, lymphocytes, monocytes, neutrophils and GFR, all with *P* < 0.001. Baseline characteristics of participants stratified by LnSII quartile are presented in Tables 1, 2.

**Table 1:** Baseline characteristics of HF patients by LnSII quartiles.

Baseline data	Total count (*n* = 1,084)	Q1 (2.36–5.84) (*n* = 271)	Q2 (5.84–6.22) (*n* = 271)	Q3 (6.22–6.66) (*n* = 271)	Q4 (6.66–8.78) (*n* = 271)	x^2^/t/Z-value	*P*-value
Age	62 (54.3,70.0)	62 (54.0,69.0)	60 (53.0,67.0)	61 (53.8,70.0)	66 (57.0,72.0)	16.591	<0.001
Gender (n,%)					8.344	0.039	
Male	853 (78.7)	220 (81.2)	224 (82.4)	211 (78.1)	198 (73.1)		
Female	231 (21.3)	51 (18.8)	48 (17.6)	59 (21.9)	73 (26.9)		
Pre-existing Conditions and Comorbidities (*n*,%)							
Smoking History	484 (44.6)	129 (47.6)	129 (47.4)	127 (47.0)	99 (36.5)	9.654	0.022
Hypertension	486 (44.8)	108 (39.9)	105 (38.6)	130 (28.1)	143 (52.8)	15.084	0.002
Type 2 Diabetes	256 (23.6)	66 (24.4)	58 (21.3)	59 (21.9)	73 (26.9)	2.997	0.392
Cardiomyopathy	228 (21.0)	59 (21.8)	64 (23.5)	59 (21.9)	46 (17.0)	3.906	0.272
Valvular Heart Disease	51 (4.7)	11 (4.1)	9 (3.3)	13 (4.8)	18 (6.6)	3.710	0.295
Myocardial Infarction	355 (32.7)	96 (35.4)	96 (35.3)	84 (31.9)	77 (28.4)	4.092	0.252
PCI	398 (36.7)	107 (39.5)	102 (37.5)	104 (38.5)	85 (31.4)	4.682	0.197
Cerebrovascular accident	64 (5.9)	12 (4.4)	14 (5.1)	9 (3.3)	29 (10.7)	15.780	0.001
Atrial fibrillation/flutter	237 (21.9)	70 (25.8)	56 (20.6)	48 (17.8)	63 (23.2)	5.697	0.127
Ventricular arrhythmia	289 (26.7)	66 (24.4)	74 (27.2)	76 (28.1)	73 (26.9)	1.095	0.778
Prior medication history (n,%)							
*β*-blockers	978 (90.2)	239 (88.2)	254 (93.4)	238 (88.1)	247 (91.1)	5.923	0.115
ACEI/ARB/ARNI	952 (87.8)	235 (86.7)	243 (89.3)	239 (88.5)	235 (86.7)	1.327	0.723
MRA	766 (70.7)	178 (65.7)	189 (69.5)	191 (70.7)	208 (76.8)	8.273	0.041
SGLT2 inhibitors	715 (66.0)	169 (62.4)	181 (66.5)	175 (64.8)	190 (70.1)	3.841	0.279
Antiplatelet agents	680 (62.7)	171 (63.1)	178 (65.4)	170 (63.0)	161 (59.4)	2.155	0.541
Anticoagulants	259 (23.9)	72 (26.6)	58 (21.3)	59 (21.9)	70 (25.8)	3.232	0.257
Heart rate^a^	78.5 (69,92)	77 (68,92)	79 (69,90)	78 (70,91.5)	86 (75,103)	24.347	<0.001
Systolic blood pressure (mmHg)^a^	123 (109,138)	120 (107,139)	124 (110,137)	122 (110,139)	123 (106,138)	2.610	0.456
Diastolic blood pressure (mmHg)^a^	78 (68,89)	76 (67,90)	80 (67.5,90)	79 (69.5,91)	77 (68,88)	2.207	0.531
LVEDV (mL)^a^	176 (141,221)	172 (145,215)	177 (136,221)	178 (129,228)	171 (136,211)	4.652	0.199
LVESV (mL)^a^	104 (77,138)	103 (82,134)	102 (72,143)	103 (71.5,140)	104 (75,134)	1.428	0.699
LVEF(%)^a^	41 (35,46)	41 (35,46)	42 (33.5,47)	41 (36,45)	39 (34,45)	7.448	0.059
HFmrEF	547 (50.5)	141 (52.0)	143 (52.8)	139 (51.3)	124 (45.8)		
HFrEF^a^	537 (49.5)	130 (48.0)	128 (47.2)	132 (48.7)	147 (54.2)		
NYHA class^a^						32.150	<0.001
I	58 (5.4)	18 (6.6)	21 (7.7)	13 (4.8)	6 (2.2)		
II	345 (31.8)	89 (32.8)	97 (35.8)	81 (29.9)	78 (28.8)		
III	435 (40.1)	125 (46.1)	96 (35.4)	110 (40.6)	104 (38.4)		
IV	246(22.7)	39(14.4)	57(21.0)	67(24.7)	83(30.6)		
BMI[(kg/m2)]^a^	24.4(22.0,26.8)	24.2(21.9,25.9)	24.2(22.0,25.8)	24.5(22.1,26.5)	24.5(21.5,27.0)	1.278	0.734

PCI, Percutaneous coronary intervention; ACEI, Angiotensin-Converting Enzyme Inhibitor; ARB, Angiotensin Receptor Blocker; ARNI, Angiotensin-N-Endopeptidase Inhibitor; MRA, Mineralocorticoid Receptor Antagonist; SGLT2i, Sodium-Glucose Transporter 2 Inhibitor; LVEDV, Left ventricular end-diastolic volume; LVESV, Left ventricular end-systolic volume; LVEF, Left ventricular ejection fraction; HErEF, Heart Failure with reduced Ejection Fraction; HEmrEF, Heart Failure with mildly reduced Ejection Fraction; BMI, Body Mass Index.

a M(Q1,Q3).

**Table 2:** Laboratory characteristics of HF patients by LnSII quartiles.

Laboratory results	Total count (*n* = 1,084)	Q1 (2.36–5.84) (*n* = 271)	Q2 (5.84–6.22) (*n* = 271)	Q3 (6.22–6.66) (*n* = 271)	Q4 (6.66–8.78) (*n* = 271)	x^2^/t/Z-value	*P*-value
NT-proBNP ((pg/mL)^a^	1,705 (582.5, 4,490)	1,510 (556, 4,000)	1,630 (547.5, 3,490)	1,280 (483, 5,705)	3,670 (1,310, 10,000)	58.323	<0.001
WBC (×10^9^/L)^a^	6.25 (5.15,7.51)	5.50 (4.19,6.75)	5.74 (4.55,6.88)	6.41 (5.43,7.86)	6.90 (5.94,8.33)	146.494	<0.001
RBC (×10^12^/L)^a^	4.81 (4.37, 5.21)	4.75 (4.31, 5.15)	4.78 (4.42, 5.16)	4.81 (4.31, 5.12)	4.64 (4.14, 5.17)	14.157	0.003
HGB (g/L)^a^	151 (137, 163)	149 (136, 163)	153 (139.5, 163.5)	151 (133.5, 160.5)	143 (126, 157)	33.718	<0.001
PLT (×10^9^/L)^a^	181 (143, 221)	139 (107, 176)	160 (135, 193)	196 (157.5, 237.5)	221 (178, 278)	299.301	<0.001
LYM (×10^9^/L)^a^	1.44 (1.05, 1.86)	1.68 (1.27, 2.31)	1.43 (1.11,,1.92)	1.43 (1.08,,1.83)	0.95 (0.73,,1.21)	195.964	<0.001
MON (×10^9^/L)^a^	0.39 (0.31,,0.50)	0.37 (0.28, 0.45)	0.38 (0.28, 0.47)	0.39 (0.31, 0.47)	0.42 (0.32, 0.57)	44.260	<0.001
NE (×10^9^/L)^a^	4.11 (3.29, 5.20)	3.24 (2.44, 3.93)	3.68 (3.01, 4.48)	4.59 (3.71, 5.32)	5.38 (4.49, 6.75)	381.352	<0.001
TC (mmol/L)^a^	3.50 (2.93, 4.24)	3.52 (2.98, 4.29)	3.32 (2.90, 4.06)	3.35 (2.78, 4.36)	3.47 (2.94, 4.12)	1.511	0.690
TG (mmol/L)^a^	1.31 (0.94, 1.87)	1.44 (0.94, 2.13)	1.40 (0.95, 1.99)	1.27 (0.89, 1.77)	1.19 (0.92, 1.68)	6.250	0.100
HDL-C (mmol/L)^a^	0.97 (0.83, 1.13)	0.94 (0.82, 1.09)	0.96 (0.83, 1.10)	0.96 (0.82, 1.14)	0.95 (0.79, 1.22)	1.047	0.790
LDL-C (mmol/L)^a^	2.19 (1.78, 2.75)	2.25 (1.81, 2.84)	2.04 (1.72, 2.66)	2.10 (1.66, 2.79)	2.21 (1.81, 2.71)	2.476	0.480
Lp (a)(g/L)^a^	14.24 (6.6, 31.38)	8.8 (5.04, 26.92)	11.89 (6.55, 26.02)	13.83 (6.49, 28.96)	16.61 (7.74, 36.63)	4.318	0.229
GFR	88.1 (76.4, 102.0)	89.9 (77.6, 102.0)	90.5(81.0, 103.0)	88.5(77.0, 103.0)	83.7(67.4, 98.6)	19.814	<0.001

NT-proBNP, N-terminal pro-B-type natriuretic peptide precursor; WBC, white blood cell count; RBC, red blood cell count; HGB, hemoglobin count; PLT, platelet count; LYM, lymphocyte count; MON, monocyte count; NE: neutrophil count; TC, total cholesterol count; TG, Triglyceride count; HDL-C, High-density lipoprotein cholesterol; LDL-C, Low-density lipoprotein cholesterol; Lp(a), Lipoprotein(a); GFR, Glomerular Filtration Rate.

a M(Q1,Q3).

### Association of LnSII with all-cause and cardiovascular mortality

During the follow-up period, a total of 142 all-cause deaths (13.1%) and 60 cardiovascular deaths (5.5%) were observed. A Cox proportional hazards model was used to assess the association between LnSII quartiles (Q1: 2.36–5.84; Q2: 5.84–6.22; Q3: 6.22–6.66; Q4: 6.66–8.78) and all-cause mortality (ACM). In models adjusted for various combinations of variables, the risk of all-cause mortality was significantly increased in the fourth quartile (Q4) (HR = 1.59, 95% CI: 1.03–2.46 in the fully adjusted Model 4), whereas the second and third quartiles (Q2, Q3) showed no statistically significant difference compared with the reference group (Q1). To further address potential residual confounders, we constructed an extended Model 4, which additionally included atrial fibrillation/flutter, prior myocardial infarction, and hemoglobin as covariates. The results indicate that elevated SII levels remain an independent predictor of all-cause mortality in patients with heart failure. To further explore the mechanisms underlying SII, this study incorporated markers reflecting cardiac function and stress (LVEF and NT-proBNP) into the adjustment models (Model 5). The HRs (95% CI) for ACM were 1.00 (reference), 0.64 (0.38–1.10), 0.92 (0.56–1.51), and 1.34 (0.86–2.09), respectively. The association between LnSII and mortality was significantly weakened, suggesting that the effect of SII on mortality risk may be partially mediated through exacerbation of cardiac dysfunction and myocardial stress, as shown in Table 3.

**Table 3:** Adjusted and unadjusted cox hazard models for LnSII and all-cause/ cardiovascular mortality.

HR (95%Cl), *P*-value					
	Model1	Model2	Model3	Model4	Model5
All-cause Mortality					
LnSII	1.64 (1.31-2.06),<0.001	1.48 (1.18, 1.85),<0.001	1.49 (1.19–1.87),<0.001	1.50 (1.20–1.88),<0.001	1.29 (1.04–1.61), 0.024
LnSII (quartile)					
Q1	Ref.	Ref.	Ref.	Ref.	Ref.
Q2	0.64 (0.37–1.10), 0.106	0.66 (0.39–1.13), 0.130	0.65 (0.38–1.11), 0.114	0.69 (0.41–1.19), 0.182	0.64 (0.38–1.10), 0.109
Q3	0.89 (0.55–1.46), 0.659	0.90 (0.55–1.46), 0.650	0.91 (0.55–1.48), 0.697	0.97 (0.59–1.59), 0.889	0.92 (0.56–1.51), 0.733
Q4	1.78 (1.16–2.73), 0.008	1.60 (1.04–2.45), 0.032	1.58 (1.03–2.44), 0.037	1.59 (1.03–2.46), 0.036	1.34 (0.86–2.09), 0.196
*P* for trend	<0.001	0.010	0.011	0.013	0.073
Cardiovascular Mortality					
LnSII	1.20 (0.83–1.74), 0.337	1.13 (0.79–1.63), 0.499	1.15 (0.80–1.66), 0.445	1.09 (0.77–1.53), 0.638	0.99 (0.70–1.38), 0.928
LnSII (quartile)					
Q1	Ref.	Ref.	Ref.	Ref.	Ref.
Q2	0.63 (0.30–1.29), 0.202	0.64 (0.31–1.32), 0.223	0.64 (0.31–1.32), 0.229	0.74 (0.36–1.54), 0.422	0.66 (0.31–1.37), 0.259
Q3	0.59 (0.28–1.23), 0.160	0.59 (0.28–1.23), 0.156	0.62 (0.29–1.31), 0.212	0.65 (0.31–1.39), 0.266	0.61 (0.29–1.30), 0.204
Q4	1.03 (0.54–1.96), 0.937	0.95 (0.50–1.82), 0.884	0.97 (0.51–1.87), 0.928	0.87 (0.44–1.69), 0.674	0.74 (0.37–1.47), 0.388
*P* for trend	0.995	0.820	0.892	0.624	0.389

Model 1: Unadjusted; Model 2: Adjusted for age and sex; Model 3: Adjusted for age, sex + hypertension, diabetes, BMI, smoking history; Model 4: Adjusted for age, sex + hypertension, diabetes, BMI, smoking history + atrial fibrillation/flutter, prior myocardial infarction, hemoglobin; Model 5: Adjusted for age, sex + hypertension, diabetes, BMI, smoking history + atrial fibrillation/flutter, prior myocardial infarction, hemoglobin + LVEF, NT-proBNP.

Internal validation using bootstrap resampling (1,000 iterations) confirmed the robustness of the association between LnSII and all-cause mortality. The bootstrap-corrected HR for Q4 vs Q1 was 1.591 (95% CI: 1.038–2.472), which was nearly identical to the original estimate (HR = 1.591, 95% CI: 1.030–2.457), indicating that the association was stable and not driven by sampling outliers.

The proportional hazards assumption was tested for all Cox models. For the primary model (Model 4), the global Schoenfeld residual test yielded a *P*-value of 0.3004 for all-cause mortality and 0.3849 for cardiovascular mortality, indicating no statistically significant violation of the proportional hazards assumption. Detailed results for models and covariates are provided in Supplementary Table S1.

### Nonlinear association between LnSII and all-cause mortality and cardiovascular disease mortality

**Figure 2:**
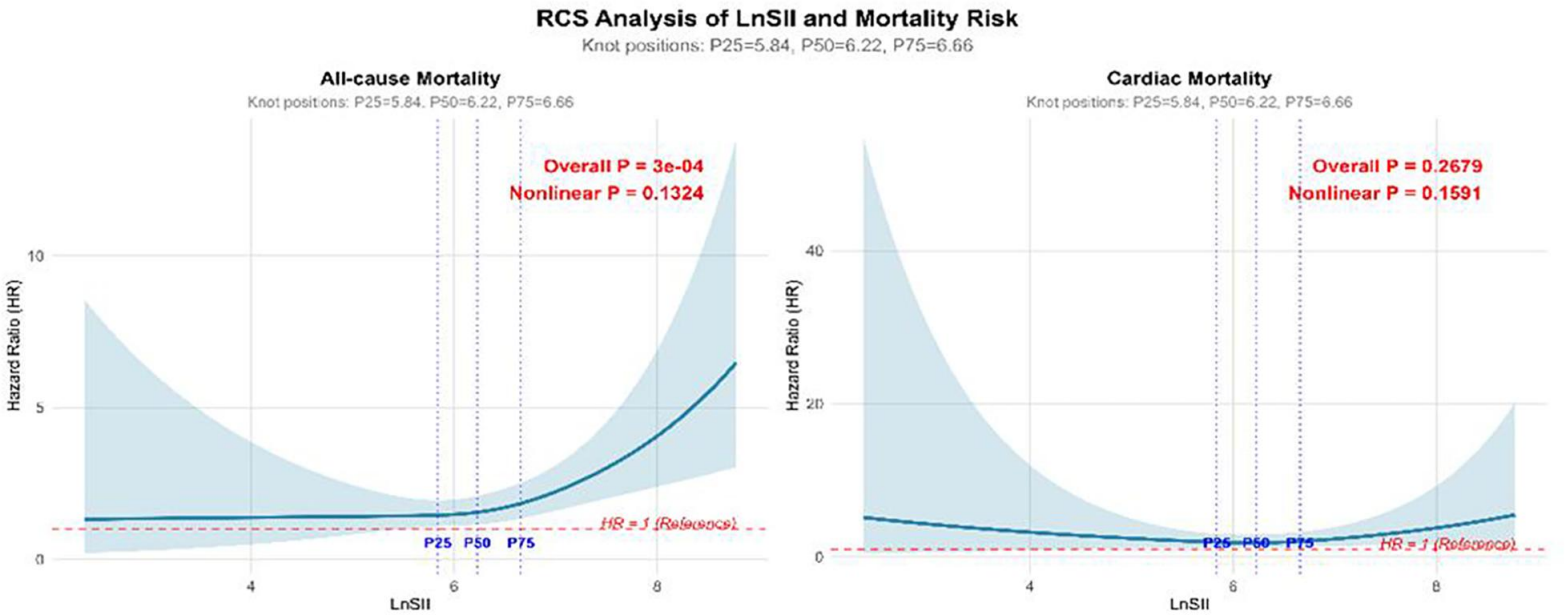
RCS analysis of LnSII and all-cause and cardiovascular mortality risk.

**Figure 3:**
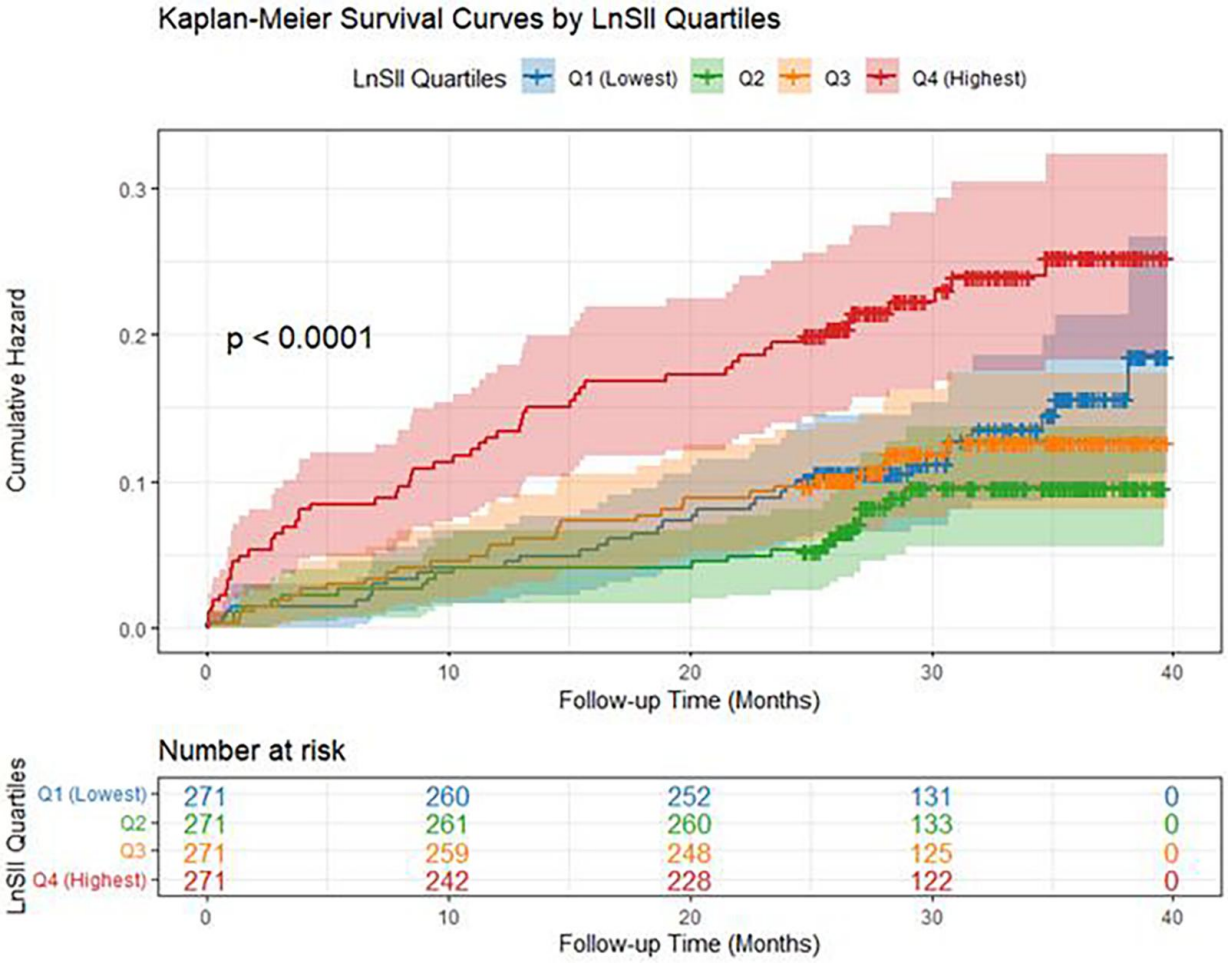
Kaplan-Meler survival curves for LnSII quartiles and all-cause mortality.

This study employed an RCS-based Cox proportional hazards model, placing three nodes at the 25th, 50th, and 75th percentiles of the LnSII distribution within the study cohort to capture potential nonlinear characteristics. In our RCS analysis, we placed knots at the 25th, 50th, and 75th percentiles of the LnSII distribution (P25 = 5.84, P50 = 6.22, P75 = 6.66). The hazard ratios are relative to the reference value at the 25th percentile (LnSII = 5.84). Solid lines represent adjusted hazard ratios; shaded areas indicate 95% confidence intervals. *P*-values for nonlinearity are shown. The *p*-values for nonlinearity were as follows: All-cause mortality(Nonlinear *P* = 0.0003), Cardiovascular mortality(Nonlinear *P* = 0.132); LnSII showed a significant positive correlation with all-cause mortality but no significant association with cardiac mortality. This suggests that the predictive role of LnSII for mortality primarily manifests in non-cardiac deaths, or its effect on cardiac mortality may be masked by other confounding factors. Kaplan–Meier survival curves demonstrated that the cumulative risk of all-cause mortality was significantly higher in the highest LnSII quartile (Q4) compared to other quartiles, as shown in Figures 2, 3.

### Subgroup analysis

Subgroup analysis explored heterogeneity in the association between LnSII and ACM/CVM. Among smokers, elevated LnSII significantly increased ACM risk (R = 2.41, 95% CI = 1.57–3.68, *P* < 0.001), with a significant interaction (*P* interactio*n* = 0.023). However, because smoking status was categorized into two groups, we were unable to distinguish between current and former smokers, nor could we quantify cumulative exposure. Among individuals with BMI ≥ 25, elevated LnSII levels substantially increased CVD risk (HR = 2.17, 95% CI = 1.14–4.12), with a significant interaction (*P* interaction = 0.038). To assess whether the association between LnSII and all-cause mortality and cardiac mortality differed by heart failure phenotype, patients were stratified into HFrEF and HFmrEF groups. The adjusted hazard ratio (HR) for all-cause mortality per unit increase in LnSII was 1.72 (95% CI: 1.33–2.23) in the HFrEF group and 1.31 (95% CI: 0.83–2.05) in the HFmrEF group, with no significant interaction (interaction *P* = 0.311). as shown in Tables 4, 5.

**Table 4:** Subgroup analysis of All-cause mortality based on Cox proportional hazards model.

Stratified analysis	*N* (%)	HR (95%Cl)	*P*-value	*P* for interaction
Total count	1,084	1.64 (1.31–2.06)	<0.001	
Gender Male	853	1.77 (1.37–2.29)	<0.001	0.223
Female	231	1.27 (0.79–2.05)	0.328	
Age ≥65 years	472	1.49 (1.14–1.95)	0.003	0.793
<65 years	612	1.61 (1.05–2.45)	0.028	
Smoking Yes	484	2.41 (1.57–3.68)	<0.001	0.023
No	600	1.35 (1.03–1.76)	0.029	
BMI≥25	451	1.80 (1.24–2.61)	0.002	0.526
BMI<25	633	1.56 (1.17–2.07)	0.003	
Hypertension Yes	486	1.65 (1.18–2.30)	0.003	0.964
No	598	1.68 (1.22–2.32)	0.002	
Diabetes Yes	256	2.03 (1.38–3.00)	<0.001	0.154
No	828	1.46 (1.10–1.93)	0.008	
HFmrEF	547	1.31 (0.83–2.05)	<0.001	0.311
HFrEF	537	1.72 (1.33–2.23)	0.244	

**Table 5:** Subgroup analysis of cardiovascular mortality based on Cox proportional hazards model.

Stratified analysis	*N* (%)	HR (95%Cl)	*P*-value	*P* for interaction
Total count	1,084	1.20 (0.83–1.74)	0.336	
Gender Male	853	1.36 (0.89–2.09)	0.155	0.216
Female	231	0.80 (0.38–1.68)	0.552	
Age ≥65 years	472	0.99 (0.64–1.55)	0.975	0.282
<65 years	612	1.51 (0.82–2.79)	0.189	
Smoking Yes	484	1.51 (0.70–3.22)	0.291	0.408
No	600	1.05 (0.69–1.60)	0.809	
BMI ≥ 25	451	2.17 (1.14–4.12)	0.018	0.038
BMI < 25	633	0.96 (0.62–1.49)	0.863	
Hypertension Yes	486	0.91 (0.49–1.68)	0.758	0.199
No	598	1.52 (0.95–2.43)	0.078	
Diabetes Yes	256	1.23 (0.63–2.39)	0.551	0.898
No	828	1.18 (0.75–1.84)	0.471	
HFmrEF	547	0.71 (0.32–1.58)	0.407	0.165
HFmEF	537	1.35 (0.90–2.02)	0.152	

### Mediation analysis

**Figure 4:**
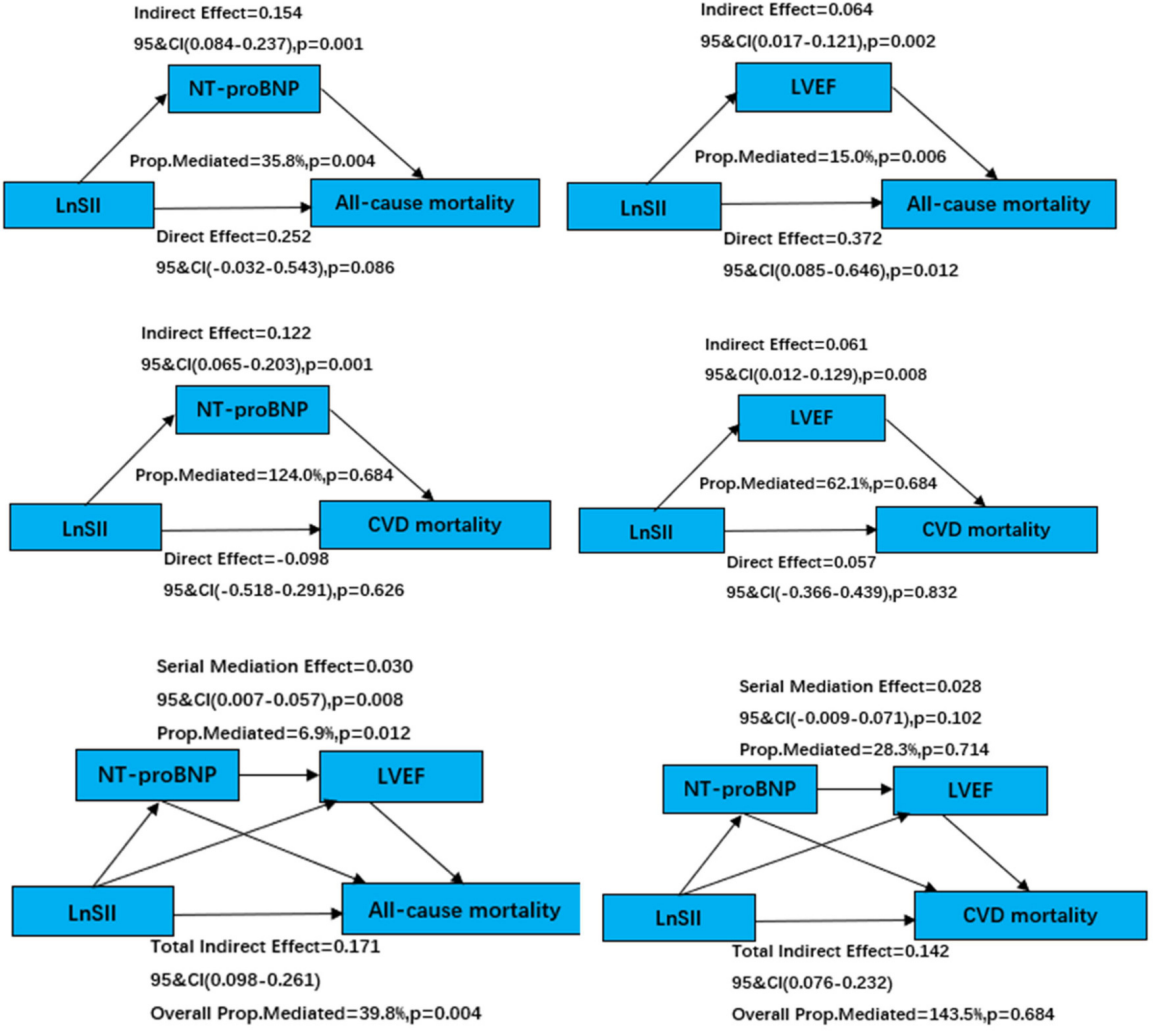
Mediation analysis of LnSII on all-cause and cardiovascular mortality.

Mediation analysis revealed that NT-proBNP and LVEF indirectly mediated the association between LnSII and ACM and CVM. NT-proBNP mediated 35.8% of the total effect of LnSII on ACM. LVEF mediated 15.0%. For all-cause mortality, the chain mediation analysis in this study revealed a significant chain mediating effect of NT-proBNP and LVEF, accounting for 6.9% of the total effect (*P* = 0.008). This finding supports a sequential pathophysiological mechanism whereby inflammation impairs cardiac function by increasing myocardial stress, ultimately leading to death. respectively, as shown in Figure 4.

### Sensitivity analysis

To further validate the robustness of the study model, sensitivity analyses were conducted. These included excluding patients who died within one week prior to follow-up. Among the 135 patients (12.5%) who experienced all-cause mortality and the 53 patients (4.9%) who experienced cardiac mortality, the results remained consistent with the primary conclusions. High SII levels remained an independent predictor of all-cause mortality in heart failure patients, as shown in Table 6.

**Table 6:** Sensitivity analysis of Cox risk models for All-cause and cardiovascular mortality in HF patients.

HR (95%Cl), *P*-value					
	Model1	Model2	Model3	Model4	Model5
All-cause Mortality					
LnSII	1.58 (1.25–2.00),<0.001	1.44 (1.14, 1.81), 0.002	1.45 (1.15–1.83), 0.001	1.45 (1.15–1.83), 0.002	1.27 (1.01–1.60), 0.040
LnSII (quartile)					
Q1	Ref.	Ref.	Ref.	Ref.	Ref.
Q2	0.63 (0.36–1.09), 0.099	0.65 (0.37–1.12), 0.120	0.64 (0.37–1.10), 0.106	0.68 (0.39–1.18), 0.171	0.63 (0.37–1.10), 0.103
Q3	0.90 (0.54–1.47), 0.655	0.90 (0.54–1.47), 0.652	0.91 (0.55–1.50), 0.718	0.98 (0.59–1.61), 0.920	0.93 (0.56–1.54), 0.771
Q4	1.71 (1.11–2.65), 0.016	1.55 (1.00–2.40), 0.052	1.54 (0.99–2.39), 0.055	1.54 (0.99–2.41), 0.056	1.32 (0.84–2.07), 0.236
*P* for trend	0.004	0.018	0.018	0.021	0.092
Cardiovascular Mortality					
LnSII	1.12 (0.76–1.64), 0.577	1.07 (0.73–1.56), 0.741	1.10 (0.75–1.60), 0.638	1.03 (0.73–1.47), 0.854	0.96 (0.68–1.35), 0.790
LnSII (quartile)					
Q1	Ref.	Ref.	Ref.	Ref.	Ref.
Q2	0.66 (0.32–1.37), 0.264	0.67 (0.32–1.39), 0.284	0.89 (0.33–1.40), 0.294	0.79 (0.38–1.66), 0.538	0.70 (0.34–1.48), 0.356
Q3	0.57 (0.26–1.22), 0.146	0.56 (0.26–1.22), 0.143	0.61 (0.28–1.32), 0.207	0.64 (0.29–1.41), 0.267	0.61 (0.28–1.33), 0.212
Q4	1.03 (0.53–1.99), 0.937	0.96 (0.50–1.88), 0.915	1.00 (0.51–1.96), 0.995	0.89 (0.45–1.77), 0.738	0.78 (0.38–1.57), 0.481
*P* for trend	0.947	0.802	0.916	0.648	0.444

Model 1: Unadjusted; Model 2: Adjusted for age and sex; Model 3: Adjusted for age, sex + hypertension, diabetes, BMI, smoking history; Model 4: Adjusted for age, sex + hypertension, diabetes, BMI, smoking history + atrial fibrillation/flutter, prior myocardial infarction, hemoglobin; Model 5: Adjusted for age, sex + hypertension, diabetes, BMI, smoking history + atrial fibrillation/flutter, prior myocardial infarction, hemoglobin + LVEF, NT-proBNP.

To account for competing risks from non-cardiovascular death, we performed sensitivity analysis using the Fine-Gray subdistribution hazard model (Supplementary Figure S1). Consistent with the primary Cox regression results, there was no significant association between LnSII and cardiovascular mortality. The subdistribution hazard ratio (SHR) for the highest versus lowest LnSII quartile (Q4 vs Q1) was 0.797 (95% CI: 0.392–1.622, *P* = 0.532), supporting the robustness of the null finding.

To assess the potential impact of differential follow-up duration, we performed sensitivity analyses adjusting for enrollment year (Supplementary Table S2). The hazard ratio for LnSII Q4 vs Q1 remained unchanged after adjustment (HR = 1.591, 95% CI: 1.030–2.457, *P* = 0.036). Enrollment year itself was not associated with all-cause mortality (HR = 0.975, 95% CI: 0.679–1.401, *P* = 0.891). Stratified analysis by enrollment year yielded consistent estimates (2022: HR = 1.620, 95% CI: 0.938–2.798; 2023: HR = 1.530, 95% CI: 0.725–3.231), with no significant interaction between LnSII and enrollment year (*P* for interaction = 0.889).

Sensitivity analyses stratified by SGLT2 inhibitor use showed that the association between LnSII and all-cause mortality was significant in patients receiving SGLT2 inhibitors (HR = 1.96, 95% CI: 1.17–3.26, *P* = 0.010), but not in those not receiving SGLT2 inhibitors (HR = 0.93, 95% CI: 0.38–2.26, *P* = 0.871). However, the interaction between LnSII and SGLT2 inhibitor use was not significant (*P* for interaction = 0.889), indicating that the prognostic value of SII was not modified by SGLT2 inhibitor treatment (Supplementary Table S3). These results support the robustness of the primary findings.

## Discussion

In this retrospective cohort of hospitalized patients with heart failure, LnSII was positively associated with subsequent all-cause mortality, whereas the association with cardiovascular mortality was not statistically significant after comprehensive adjustment, which may be attributable to the limited number of cardiovascular deaths (*n* = 60) and consequent reduced statistical power. This pattern, together with the absence of an obvious non-linear relationship, supports a graded prognostic signal whereby a higher immune–inflammatory burden, as captured by LnSII, corresponds to a higher overall risk of death. Importantly, the attenuation of the association after incorporating markers of cardiac function and hemodynamic stress, along with the observed indirect effects through NT-proBNP and LVEF, suggests that LnSII may partly operate through pathways linked to disease severity and systemic inflammatory activation rather than acting as an isolated risk factor. Furthermore, the mediation analysis was conducted using cross-sectional baseline data, in which SII, NT-proBNP, and LVEF were measured simultaneously. Therefore, it is not possible to establish a clear temporal sequence—that is, that exposure precedes the mediators, and the mediators precede the outcome. Although the observed indirect effects are consistent with the mediating roles of NT-proBNP and LVEF, reverse causality cannot be ruled out (i.e., more severe heart failure leading to elevated SII). Therefore, these findings should be considered exploratory and used to generate hypotheses; future studies utilizing longitudinal data are needed to rigorously assess causal pathways.

The present results are consistent with contemporary views that inflammation is not merely an epiphenomenon but a central component in the pathobiology of heart failure, involving both innate and adaptive immune activation, cytokine signaling, endothelial dysfunction and remodeling processes (6, 7). Recent syntheses further highlight that myocardial inflammation is present across both HFrEF and HFpEF, while the cardioimmunology interface may shape phenotypic heterogeneity and outcome trajectories (8, 9). In HFpEF, inflammatory pathways driven by comorbidities and extracardiac sources have been emphasized as major upstream determinants of microvascular dysfunction, fibrosis and impaired relaxation, providing a plausible substrate for why systemic immune–inflammatory indices may retain prognostic meaning in real-world cohorts (10). In parallel, the balance between inflammatory activation and resolution signaling has been recognized as critical for cardiac repair and the transition toward chronic dysfunction, reinforcing the concept that persistent low-grade inflammation may confer long-term risk (11). Within this framework, a composite index integrating neutrophils, platelets and lymphocytes could reasonably summarize concurrent innate immune activation, thrombo-inflammatory propensity and relative adaptive immune suppression—features frequently observed in advanced heart failure.

The association between SII-related metrics and adverse outcomes has been reported in multiple heart failure settings. In advanced chronic heart failure patients with renal dysfunction, higher SII has been proposed as a prognostic marker, supporting the link between systemic inflammation and vulnerability in high-risk phenotypes (12). In acute decompensated heart failure, the prognostic value of SII has also been evaluated, further suggesting that immune–inflammatory burden conveys risk information beyond traditional clinical variables (13). Population-based evidence has shown associations between higher SII and heart failure status in large survey cohorts, implying that systemic immune activation is intertwined with heart failure prevalence at the population level (4). In HFpEF specifically, SII has been associated with adverse outcomes, reinforcing the relevance of immune–inflammatory profiling in this phenotype (14). Moreover, hematologic inflammatory ratios closely related to SII biology (neutrophil-to-lymphocyte and platelet-to-lymphocyte ratios) have demonstrated prognostic utility for post-discharge cardiac death in acute HFpEF, lending additional support to the concept that leukocyte/platelet–lymphocyte balance reflects clinically meaningful risk (15). Taken together, these studies provide convergent evidence that LnSII, as a log-transformed derivative of SII, can serve as a pragmatic risk marker in heart failure populations.

Biological plausibility is further strengthened by emerging mechanistic data on immune dysregulation in heart failure. Contemporary translational work indicates that immune-cell programs and myocardial inflammation differ across HFrEF and HFpEF, yet both phenotypes share overlapping immune-mediated injury and remodeling signals (8, 9). The “systemic comorbidity–inflammation” axis emphasized in HFpEF aligns with the observation that systemic indices such as SII/LnSII may capture extracardiac inflammatory drivers that feed into cardiac dysfunction (10). Experimental evidence also supports a direct pathogenic role of adaptive immune dysfunction in HFpEF: impaired T-cell IRE1*α*/XBP1 signaling has been shown to direct inflammation and contribute to HFpEF features, highlighting that immune pathway derangements can be mechanistically upstream of cardiac dysfunction rather than downstream consequences alone (16). These findings offer a mechanistic rationale for interpreting LnSII as more than a passive biomarker, particularly when considered alongside mediating signals through NT-proBNP and LVEF.

The present mediation findings are also coherent with established cytokine networks in heart failure. In patients recently hospitalized with HFpEF, IL-6 has been reported as an independent predictor of all-cause and cardiovascular outcomes even after adjustment for natriuretic peptides, suggesting that inflammatory signaling provides additive prognostic information beyond congestion-related biomarkers (17). Complementary work in HFpEF populations further characterizes IL-6-related risk and its association with comorbidity burden, supporting the biological coherence between systemic inflammation, cardiometabolic stress and clinical outcomes (18). These observations are aligned with the therapeutic interest in anti-inflammatory strategies. IL-1 blockade studies have provided signals relevant to functional status in heart failure, and trial designs such as REDHART2 illustrate continued efforts to test inflammatory pathway inhibition in recently decompensated heart failure using randomized methodologies (19, 20). Beyond cytokine blockade, leukocyte immunomodulation has been proposed as a broader strategy to target systemic inflammation in heart failure (21). Given the expanding role of cardiometabolic therapies, perspectives also emphasize that important knowledge gaps remain regarding SGLT2 inhibitors and GLP-1 receptor agonists, which is relevant because metabolic dysregulation and inflammation often coexist in heart failure phenotypes (22). An exploratory analysis of DAPA-HF has specifically examined IL-6 in HFrEF and the effect of dapagliflozin, providing an additional rationale to consider interactions between anti-congestion/anti-remodeling therapies and inflammatory biology (23). Nevertheless, guideline-based foundational therapy—particularly in ischemic etiologies—remains central, and any biomarker-driven approach should be integrated within established treatment frameworks rather than used in isolation (24).

Subgroup findings suggesting stronger associations of LnSII with outcomes among smokers and patients with higher BMI may reflect heterogeneity in baseline inflammatory tone and cardiometabolic susceptibility. Smoking and obesity can amplify systemic inflammation and endothelial dysfunction, potentially strengthening the linkage between immune–inflammatory burden and adverse clinical trajectories. This interpretation is consistent with HFpEF-focused literature emphasizing comorbidity-driven inflammatory pathways and the relevance of inflammatory endotypes in determining prognosis and therapeutic responsiveness (10, 17, 18). Therefore, LnSII may be particularly useful for identifying subgroups in whom inflammatory activity and cardiometabolic stress interact to confer disproportionate risk. The prognostic value of LnSII was consistent across different HF phenotypes (HFrEF, HFmrEF) in our subgroup analyses, suggesting that systemic inflammation is a common risk factor across the spectrum of heart failure. However, we cannot exclude the possibility that the relationship between SII and mortality may differ between patients admitted with acute decompensation and those admitted for chronic management. While our sensitivity analyses using NT-proBNP as a proxy for disease severity suggested robustness, future studies with detailed admission phenotyping are needed to confirm these findings.

Clinically, LnSII has practical advantages: it is derived from routine blood counts, incurs minimal additional cost, and can be readily applied at admission to support risk stratification. Its observed association with all-cause mortality suggests potential utility for identifying patients who warrant closer surveillance, intensified management of comorbidities, and consideration of inflammation—targeted strategies as evidence evolves (6, 19–21).

Several limitations should be acknowledged. The retrospective single—center design limits causal inference and generalizability, and residual confounding is possible. Although an external validation cohort was not available, internal bootstrap resampling demonstrated that the association between LnSII and all—cause mortality was robust to sampling variability (bootstrap—corrected HR = 1.591, 95% CI: 1.038–2.472).

Cardiovascular mortality may be particularly vulnerable to limited event numbers and competing risks, which could partially explain the null finding after full adjustment. Patients who died during hospitalization or were admitted with acute myocardial infarction were excluded from this analysis. While this exclusion was necessary to assess post—discharge long—term outcomes, it may have introduced selection bias by removing individuals with the most severe illness and the highest inflammatory burden. Consequently, the association between SII and cardiovascular mortality may be underestimated in our study, and our findings should be interpreted as applicable primarily to patients who survive the acute phase of heart failure without concurrent acute myocardial infarction.

Future studies including these high—risk populations are needed to fully characterize the prognostic value of SII across the full spectrum of heart failure severity. LVEF was assessed only at baseline, which may not capture its dynamic changes during follow—up. A single measurement cannot distinguish between patients with transient versus persistent systolic dysfunction. Therefore, our findings should be interpreted in the context of baseline clinical status.

Future studies with serial echocardiographic assessments are warranted to evaluate the interplay between systemic inflammation, LVEF dynamics, and long—term outcomes. Additionally, LnSII was assessed at baseline; dynamic changes in inflammatory status over time were not captured, and longitudinal measurement might improve prognostic characterization.

These results support the reliability of our single—center findings, while acknowledging that external validation in diverse populations remains necessary to establish generalizability. Future multicenter prospective studies with repeated biomarker assessments and adjudicated causes of death are warranted to validate these findings and to clarify whether LnSII offers incremental predictive value across heart failure phenotypes and therapeutic backgrounds (7, 8, 10, 14, 16).

## Data Availability

The raw data supporting the conclusions of this article will be made available by the authors, without undue reservation.
