# Correction: Association between systemic immune-inflammation index(SII) and all-cause and cardiovascular mortality in heart failure patients: a single-center retrospective analysis

**DOI:** 10.3389/fcvm.2026.1874587

**Published:** 2026-05-27

**Authors:** Junlan Zhang, Nan Lyu, Yaping Zhang, Pan Chen, Qianyu Ma, Nana Hu, Bingxin Men, Xiaolei Shi, Changsen Wang, Jin Zhang

**Affiliations:** 1 Lanzhou University First Hospital, Lanzhou, China; 2 Wenzhou Medical University School of Public Health, Wenzhou, China; 3 Anhui Medical University Affiliated Fuyang People’s Hospital, Fuyang, China

**Keywords:** all-cause mortality, cardiovascular mortality, heart failure, nflammation, systemic immune-inflammatory index (SII)

Authors Nan Lyu and Yaping Zhang were erroneously omitted as equal contributing authors. The original version of this article has been updated.

