# Supplementary material for: Association between systemic immune-inflammation index(SII) and all-cause and cardiovascular mortality in heart failure patients: a single-center retrospective analysis
Source: Front Cardiovasc Med. 2026 Apr 24;13:1823641. doi: 10.3389/fcvm.2026.1823641 (PMC13154384; doi:10.3389/fcvm.2026.1823641)
Supplement: Supplementary file 1 [file Table1.docx]

| Table S1 The proportional hazards assumption | | | | | | |
| --- | --- | --- | --- | --- | --- | --- |
| Outcome | Model | Variable | P-value | Model | Variable | P-value |
| All-cause mortality | **Model 4** | **GLOBAL** | 0.3004 | **Model 5** | **GLOBAL** | 0.3535 |
|  |  | LnSII | 0.0024 |  | LnSII | 0.0041 |
|  |  | Age | 0.7563 |  | Age | 0.7376 |
|  |  | Sex | 0.5462 |  | Sex | 0.5349 |
|  |  | Hypertension | 0.5394 |  | Hypertension | 0.4996 |
|  |  | Diabetes | 0.1982 |  | Diabetes | 0.1584 |
|  |  | BMI | 0.8160 |  | BMI | 0.7966 |
|  |  | Smoking | 0.8411 |  | Smoking | 0.8529 |
|  |  | Prior-MI | 0.0745 |  | Prior-MI | 0.0724 |
|  |  | AF | 0.3145 |  | AF | 0.3792 |
|  |  | HGB | 0.4739 |  | HGB | 0.4499 |
|  |  |  |  |  | LVEF | 0.7649 |
|  |  |  |  |  | NT-proBNP | 0.1871 |
|  | **Model 4** | **GLOBAL** | 0.3849 | **Model 5** | **GLOBAL** | 0.5869 |
| Cardiovascular mortality |  | LnSII | 0.0125 |  | LnSII | 0.0191 |
|  |  | Age | 0.6570 |  | Age | 0.6621 |
|  |  | Sex | 0.7810 |  | Sex | 0.7656 |
|  |  | Hypertension | 0.1099 |  | Hypertension | 0.0959 |
|  |  | Diabetes | 0.3988 |  | Diabetes | 0.3712 |
|  |  | BMI | 0.9420 |  | BMI | 0.9232 |
|  |  | Smoking | 0.9211 |  | Smoking | 0.9291 |
|  |  | Prior-MI | 0.1414 |  | Prior-MI | 0.1396 |
|  |  | AF | 0.9095 |  | AF | 0.8895 |
|  |  | HGB | 0.6025 |  | HGB | 0.5806 |
|  |  |  |  |  | LVEF | 0.6522 |
|  |  |  |  |  | NT-proBNP | 0.5524 |
