# Supplementary material for: Association between systemic immune-inflammation index(SII) and all-cause and cardiovascular mortality in heart failure patients: a single-center retrospective analysis
Source: Front Cardiovasc Med. 2026 Apr 24;13:1823641. doi: 10.3389/fcvm.2026.1823641 (PMC13154384; doi:10.3389/fcvm.2026.1823641)
Supplement: Supplementary file 2 [file Table2.docx]

| Table S2. Sensitivity Analysis for Enrollment Period | | | |
| --- | --- | --- | --- |
| Model | Variable | HR (95% CI) | P value |
| Primary model (Model 4) | LnSII Q4 vs Q1 | 1.59(1.03-2.46) | 0.036 |
| Sensitivity model (adjusted for enrollment year) | LnSII Q4 vs Q1 | 1.59(1.03 - 2.46) | 0.036 |
|  | Enrollment year (2023 vs 2022) | 0.98(0.68–1.40) | 0.891 |
| Stratified analysis |  |  |  |
| Enrolled in 2022 (n = [608]) | LnSII Q4 vs Q1 | 1.62(0.94 - 2.80) | 0.082 |
| Enrolled in 2023 (n = [476]) | LnSII Q4 vs Q1 | 1.53(0.73 - 3.23) | 0.264 |
|  |  |  |  |
| Interaction test | LnSII Q4 × enrollment year |  | 0.889 |
| *Notes: Model 4 was adjusted for age, sex, hypertension, diabetes, BMI, smoking history, atrial fibrillation/flutter, prior myocardial infarction, and hemoglobin. Enrollment year was defined as the year of hospital discharge (2022 vs 2023).* | | | |
