# Supplementary material for: Association between systemic immune-inflammation index(SII) and all-cause and cardiovascular mortality in heart failure patients: a single-center retrospective analysis
Source: Front Cardiovasc Med. 2026 Apr 24;13:1823641. doi: 10.3389/fcvm.2026.1823641 (PMC13154384; doi:10.3389/fcvm.2026.1823641)
Supplement: Supplementary file 3 [file Table3.docx]

| **Table S3. Sensitivity Analysis for SGLT2 inhibitor** | | | |
| --- | --- | --- | --- |
| **Model** | Variable | HR (95% CI) | P value |
| **Primary model (Model 4)** | LnSII Q4 vs Q1 | 1.59(1.03-2.46) | 0.036 |
| **Sensitivity model (adjusted for SGLT2 inhibitor)** | LnSII Q4 vs Q1 | 2.00(0.75-5.35) | 0.167 |
| **Stratified analysis** |  |  |  |
| **No SGLT2i (n = [369])** | LnSII Q4 vs Q1 | 0.93 (0.38–2.26) | 0.871 |
| **SGLT2i (n = [715])** | LnSII Q4 vs Q1 | 1.96 (1.17–3.26) | 0.010 |
|  |  |  |  |
| **Interaction test** | LnSII Q4 ×SGLT2 inhibitor |  | 0.889 |
| *Notes: Model 4 was adjusted for age, sex, hypertension, diabetes, BMI, smoking history, atrial fibrillation/flutter, prior myocardial infarction, and hemoglobin. | | | |
